# Transcriptomic Study of Pseudo-mosaic and Uniform Aphidicolin-Treated Mouse Embryos Reveals Divergent Pathway Enrichment Profiles and Suggests Compensatory Mechanisms

**DOI:** 10.7759/cureus.117766

**Published:** 2026-10-11

**Authors:** Elaine de Quadros, Leonardo de Quadros, Julien Kann, Minglei Bian

**Affiliations:** 1 Reproductive Clinical Sciences, Old Dominion University, Norfolk, USA; 2 Medicine, Edward Via College of Osteopathic Medicine, Spartanburg, USA; 3 Biostatistics, State University of New York at Buffalo, Buffalo, USA

**Keywords:** aphidicolin, fragile sites, mosaicism, pre-implantation embryos, reproductive medicine, segmental aneuploidy, self-correction

## Abstract

Purpose

This study provides preliminary transcriptomic insights into the divergent responses of pseudo-mosaic and uniform aphidicolin-treated embryos, highlighting the need for further mechanistic studies and single-embryo-level analysis to validate these observations.

Methods

Following a preliminary experiment evaluating prolonged aphidicolin exposure, an independent cohort of 90 four-cell cryopreserved mouse embryos was used for the main experiment and randomly allocated to three groups (n = 30 each). Control embryos received no intervention. In the pseudo-mosaic group, two blastomeres were removed from each embryo, exposed to 1.4 µM aphidicolin for four hours, and reinjected into their corresponding embryo. In the uniform aphidicolin-treated group, intact embryos were exposed to 1.4 µM aphidicolin for four hours. Blastocyst formation was assessed, and RNA sequencing was performed to compare differential gene expression and pathway enrichment among the three groups.

Results

In the initial study, prolonged exposure (16 hours) to 1.4 µM aphidicolin resulted in complete cleavage-stage arrest in the uniform aphidicolin-treated group. Following four hours of aphidicolin exposure in the main study, blastocyst formation rates were not significantly different among groups: control, 26/30 (87%); pseudo-mosaic, 22/30 (73%); and uniform aphidicolin-treated, 27/30 (90%) (p > 0.05). When the control group was compared with the uniform aphidicolin-treated group, 33 genes were differentially expressed at an adjusted p-value of <0.05. When the control group was compared with the pseudo-mosaic group, 791 genes were differentially expressed at an adjusted p-value of <0.05. Gene set enrichment analysis comparing uniform aphidicolin-treated and pseudo-mosaic embryos identified divergent pathway enrichment profiles, ranging from cell-cycle and transcriptional regulation in the former group to cell-fate reprogramming and metabolic adaptation in the latter.

Conclusions

This study reveals distinct transcriptomic responses to aphidicolin-induced replication stress in pseudo-mosaic versus uniform aphidicolin-treated embryos. The findings suggest that embryos containing discordant cell populations may engage compensatory transcriptional programs that differ from those activated following uniform cellular stress. While the observed pathway profiles are consistent with DNA-damage, stress-response, metabolic, and apoptotic processes, the absence of genomic validation limits interpretation. Future studies incorporating single-embryo analysis and genomic characterization are needed to confirm these preliminary observations.

## Introduction

Aneuploidy is frequently observed in pre-implantation embryos, yet the exact origins and underlying mechanisms remain largely undefined. This is especially true for segmental or subchromosomal abnormalities. This conceptual gap largely stems from the technical challenges associated with studying early human embryonic development. Ethical considerations and limited access to human embryos for research have hindered the ability to investigate aneuploidy during the earliest stages of embryogenesis. Additionally, the molecular processes responsible for chromosome missegregation and structural rearrangements that lead to whole-chromosome and segmental aneuploidy are not thoroughly defined. While factors such as maternal age, spindle assembly checkpoint efficiency, and chromosomal cohesion are known to play a role, their interactions within the early embryo are still not fully elucidated [1-3]. Advancements in genotyping and sequencing techniques are beginning to overcome these challenges, providing new insights into the mechanisms of chromosomal instability and paving the way for improved diagnostic and therapeutic approaches in reproductive medicine.

Aneuploidy resulting from chromosome segregation errors is a significant contributor to implantation failure and pregnancy loss [4]. Nonetheless, a considerable number of chromosomally abnormal embryos can progress to the blastocyst stage, preventing their detection by morphological assessment alone [5]. Distinct mechanisms within early embryos may work to facilitate this seemingly normal development despite underlying genotypic and transcriptomic abnormalities [6]. These findings underscore the complexity of the early embryonic mechanisms that allow for implantation, fetal development, and live birth despite the presence of chromosomal aberrations.

Understanding whole chromosome abnormalities is challenging, but the mechanisms behind mosaicism and segmental aneuploidies are even more elusive. Mosaicism, in which abnormal and normal cell lines coexist, is poorly understood and represents an area of scientific uncertainty regarding the developmental potential of preimplantation embryos. Despite this, current preimplantation genetic testing for aneuploidy (PGT-A) models classify mosaicism, and clinics often use this information in patient care decisions. Preliminary data suggest that some mosaic embryos may possess mechanisms enabling normal development, including compartmentalization of abnormal cells, selective apoptosis of mutant cells, compensation by euploid cells, or the presence of reciprocal chromosomal errors that do not alter gene expression [7-9]. These findings align with data showing that chromosomal mosaicism is detected in less than 2% of chorionic villus samples, with even fewer cases subsequently confirmed in fetal tissues [10].

Mosaic aneuploidies are primarily attributed to post-zygotic mitotic errors or rescue mechanisms following meiotic errors [11]. Most errors occur within the first three mitotic divisions post-fertilization and persist through development [12]. However, the ratio of aneuploid to euploid cells generally declines between the cleavage and blastocyst stages [13-15]. Mammalian embryos also experience replication stress at fragile sites, contributing to chromosomal instability and aneuploidy [16]. In a previous large multicenter analysis of 2,066 human blastocysts undergoing PGT-A, de Quadros et al. demonstrated that segmental aneuploidy breakpoints occur significantly more frequently within known common fragile sites of the human genome than expected by chance, particularly for telomeric breaks [17]. These findings suggest that fragile genomic regions susceptible to replication stress may represent intrinsic hotspots for chromosomal instability during preimplantation development. However, while the genomic localization of these breakpoints was established, the downstream transcriptional consequences of replication stress and the embryonic response to such insults remain poorly understood.

Aphidicolin, a tetracyclic diterpenoid antibiotic, inhibits replicative DNA polymerases and disrupts DNA replication, thereby inducing replication stress [18]. Replication-fork stalling can activate ATR-mediated checkpoint signaling and alter DNA synthesis rates, while persistent replication stress can contribute to chromosome fragility and structural abnormalities [19-22]. Aphidicolin has also been used experimentally in mouse embryos to disrupt cell-cycle progression and development [23], and comparative mapping studies have demonstrated fragile sites in both human and murine genomes [24]. Aphidicolin has therefore been used experimentally to model replication stress and fragile-site instability. However, this pharmacologic model does not reproduce the full complexity of naturally occurring embryonic aneuploidy or mosaicism, which frequently arise through chromosome-segregation errors. Results obtained using aphidicolin must therefore be interpreted as responses to experimentally induced replication stress rather than direct evidence of naturally occurring embryonic aneuploidy.

Embryos have been proposed to exhibit cellular responses that may mitigate the developmental consequences of chromosomal abnormalities. These responses may include selective depletion or apoptosis of abnormal cells, differential lineage allocation, and compensatory proliferation of unaffected cells. Mouse models of chromosome mosaicism have demonstrated lineage-specific depletion of aneuploid cells with preservation of developmental potential [9]. In humans, a prospective non-selection trial reported comparable live-birth and miscarriage rates among euploid and low- to medium-grade mosaic blastocysts [25]. Human gastruloid studies have also demonstrated preferential depletion of aneuploid cells through apoptosis, with euploid cells capable of compensating for developmental deficits [26]. However, findings from experimental mouse models and human gastruloids cannot be assumed to reflect equivalent mechanisms in intact human preimplantation embryos, and the extent to which these processes contribute to normal human development remains uncertain.

Transcriptomic analysis of embryos cultured under controlled stress may help identify regulatory genes and signaling pathways associated with adaptation to developmental challenges. RNA sequencing permits high-resolution characterization of gene expression patterns, while mouse embryos provide a tractable experimental system for examining early developmental responses. The present study therefore compared the transcriptomic responses of mouse embryos subjected to uniform aphidicolin-induced replication stress with those of a pseudo-mosaic model in which replication stress was spatially restricted to a subset of blastomeres. The primary objective was to determine whether these two patterns of experimentally induced replication stress produce distinct gene expression and pathway-enrichment profiles. Because chromosomal abnormalities were not directly characterized, the pseudo-mosaic model is intended to represent cellular discordance within the embryo rather than confirmed naturally occurring chromosomal mosaicism.

## Materials and methods

Induction of DNA breaks to simulate genomic instability

Preliminary Aphidicolin Exposure Study

Initially, a preliminary study was performed to evaluate the effect of aphidicolin exposure on frozen/thawed mouse embryos and assess whether sufficient RNA could be recovered from limited sample quantities for transcriptomic analysis. Exploratory transcriptomic analysis was performed on this preliminary cohort; however, because biological replicates were not available, inferential statistical analysis of differential gene expression was not performed. The preliminary experiment was therefore considered exploratory and was not included in the replicated differential gene expression analysis of the main experiment.

Aphidicolin working medium was prepared from a 30 mM InSolution™ Aphidicolin stock solution in DMSO (MilliporeSigma) by dilution in embryo handling medium to a nominal final concentration of 1.4 µM. The laboratory preparation record describes 4.67 µL of 30 mM aphidicolin stock added to approximately 100 mL of handling medium. Using C1V1=C2V2, this preparation yields approximately 1.40 µM aphidicolin. Embryos or isolated blastomeres assigned to aphidicolin treatment were exposed to a medium containing 1.4 µM aphidicolin for the indicated treatment period (Figure 1).

**Figure 1:**
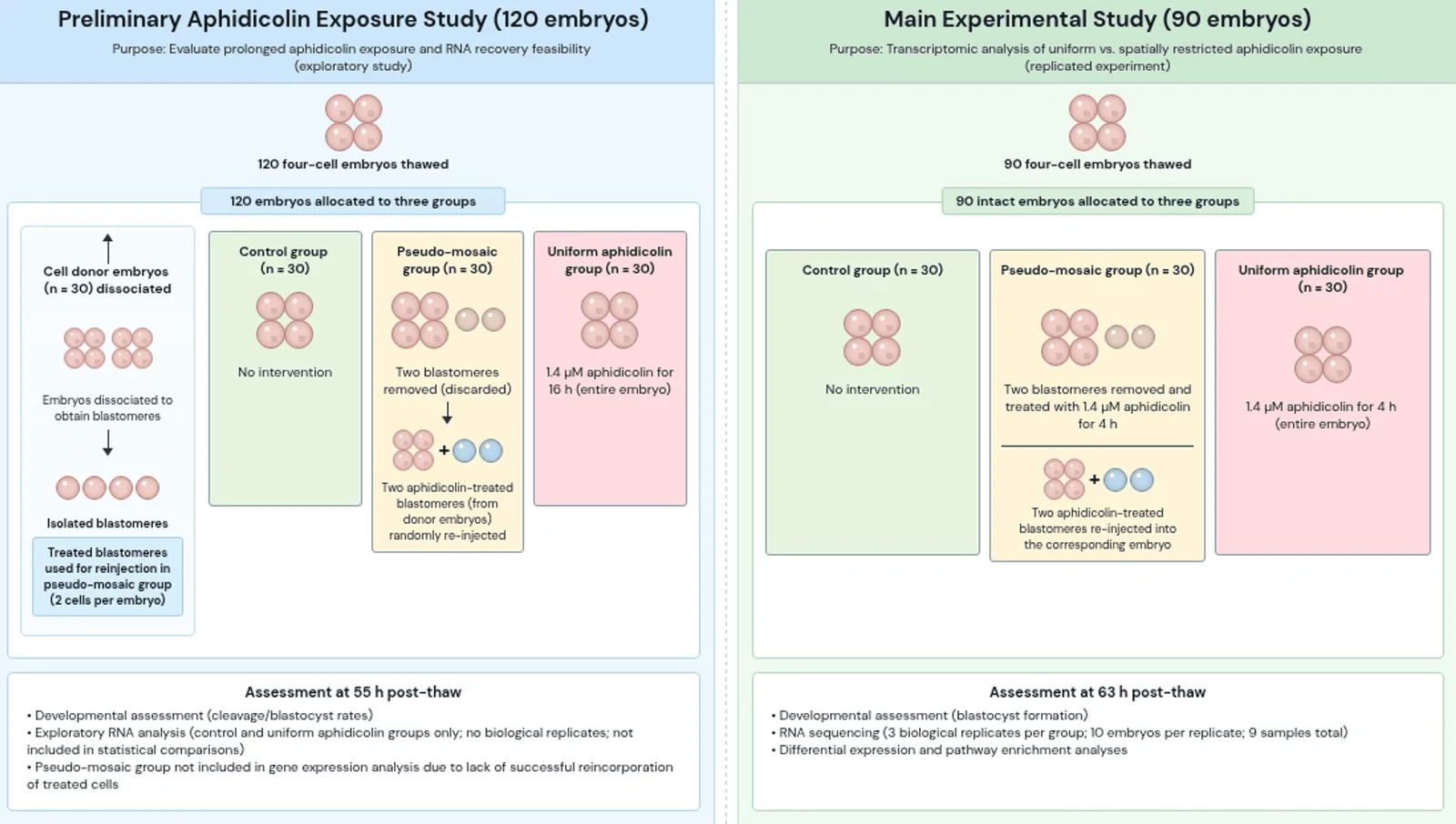
Experimental design and embryo allocation. Image Credits: This figure was created using Canva (Canva, Inc., Sydney, Australia).

A total of 120 four-cell cryopreserved mouse embryos (Embryotech Laboratories, Inc.) were thawed for the preliminary experiment. Thirty embryos were designated as cell-donor embryos and were dissociated to provide isolated blastomeres for aphidicolin treatment. The remaining 90 intact embryos were randomly stratified into three experimental groups (n = 30 per group). The control group received no intervention. For the pseudo-mosaic group, two blastomeres were removed from each of the 30 recipient embryos and discarded. Isolated blastomeres obtained from the 30 donor embryos were exposed to an M2 medium containing 1.4 µM aphidicolin for 16 hours, after which two treated blastomeres were randomly selected and reinjected into each recipient embryo. Because the treated donor blastomeres were assigned randomly rather than returned to their embryo of origin, the preliminary pseudo-mosaic manipulation differed from that used in the subsequent main experiment. The uniform aphidicolin-treated group consisted of 30 intact embryos cultured in an M2 medium containing 1.4 µM aphidicolin for 16 hours.

Following the respective treatments, embryos were rinsed and cultured individually in a time-lapse incubator (EmbryoScope+, Vitrolife Group) to the blastocyst stage. Developmental assessment was performed at 55 hours following initial incubation. Treated blastomeres in the preliminary pseudo-mosaic group did not successfully reincorporate into the recipient embryos; therefore, this group was not included in the exploratory gene expression analysis. Exploratory RNA analysis was limited to the control and uniform aphidicolin-treated groups and was not included in the replicated differential gene expression analysis of the main experiment.

This preliminary experiment was exploratory and was conducted to evaluate the effects of prolonged aphidicolin exposure and the feasibility of RNA recovery from limited embryo material. Because it differed from the main experiment in exposure duration, assessment time point, experimental groups, and analytical design, the preliminary and main experiments were treated as independent experimental cohorts, and their transcriptomic data were not pooled or directly statistically compared.

Main Experimental Study: Uniform and Spatially Restricted Aphidicolin Exposure

For the main trial, a total of 90 four-cell cryopreserved mouse embryos (Embryotech Laboratories, Inc.) were thawed and randomly stratified into three groups. The first group (n = 30) received no intervention and served as the control. In the second group (n = 30), two blastomeres were removed from each embryo and cultured in an M2 medium containing 1.4 µM aphidicolin for four hours. These treated blastomeres were then reinjected into the perivitelline space of their corresponding embryos, representing the pseudo-mosaic group. In the third group (n = 30), intact embryos were cultured in an M2 medium containing 1.4 µM aphidicolin for four hours, representing the uniform aphidicolin-treated group.

Following the respective exposure protocols, embryos were rinsed and cultured individually in a time-lapse incubator (EmbryoScope+, Vitrolife Group) to the blastocyst stage. Developmental assessment was performed at 63 hours following initial incubation. 

RNA isolation and sequencing

Total RNA was isolated using the Direct-zol RNA MiniPrep kit (Zymo Research Corp.) and treated with DNase I according to the manufacturer’s instructions. For the main experiment, 10 blastocysts were pooled to generate each biological replicate. Three independent biological replicates were generated for each experimental group, resulting in nine RNA samples in total: Group 1-A, Group 1-B, and Group 1-C (control); Group 2-A, Group 2-B, and Group 2-C (pseudo-mosaic); and Group 3-A, Group 3-B, and Group 3-C (uniform aphidicolin-treated).

RNA quality and concentration were assessed prior to library preparation using high-sensitivity RNA ScreenTape (Agilent Technologies). All nine biological replicate samples were evaluated, with measured RNA concentrations ranging from 48.9 to 119 pg/µL. Because the low-input RNA concentrations were outside the functional range for reliable RNA Integrity Number equivalent (RINe) determination, RINe values were not reported.

Libraries were prepared using low-input Illumina RNA-Seq Library Prep and sequenced on an Illumina NovaSeq X platform using paired-end 150-bp sequencing, with a target depth of approximately 50 million reads per sample. Post-sequencing sample-specific Q30 and alignment-rate metrics for the nine-sample main experiment were not available for retrospective reporting and therefore were not included.

Adapter trimming was performed using fastp. Trimmed FASTQ files were aligned to the Mus musculus reference genome (mm10) using STAR, and featureCounts was used to quantify mapped reads assigned to genomic features. Differential gene expression analysis was performed using DESeq2. Gene Ontology (GO) enrichment analysis and Gene Set Enrichment Analysis (GSEA) were performed using ShinyGO v0.81, and data visualization and graphing were performed using SRplot [27]. For all tables and figures, Groups 1, 2, and 3 represent the control, pseudo-mosaic, and uniform aphidicolin-treated groups, respectively.

Statistical analysis

Initial sample-size estimation for the RNA-sequencing experiment was based on a two-group RNA-seq sample-size framework incorporating an expected transcript count (μ) of 20, coefficient of variation of 0.60, significance level of α = 0.05, statistical power of 0.80, and a target detectable fold change of 2.2. Under these assumptions, the calculation yielded an estimated requirement of 10 samples per group. Given the limited RNA yield obtainable from individual blastocysts, 10 blastocysts were pooled to generate each biological replicate. Three independent pooled biological replicates were subsequently generated per experimental group and used for RNA-sequencing analysis. Because the original sample-size estimate was not a formal power calculation for three pooled biological replicates per group, the transcriptomic analyses were considered exploratory and hypothesis-generating.

Odds ratios (ORs) were calculated for comparisons of blastocyst formation rates between groups. Differential gene expression analysis was performed using DESeq2. Genes with a false discovery rate (FDR)-adjusted p-value ≤ 0.05 were considered differentially expressed. Log2 fold changes were calculated and reported for individual genes but were not used as an additional threshold for defining differential expression. A more stringent FDR-adjusted p-value threshold of ≤0.01 was also reported descriptively.

GO and pathway-enrichment analyses were performed using ShinyGO v0.81. Unless otherwise specified, statistical significance was evaluated using an FDR-adjusted p-value ≤ 0.05.

## Results

Blastocyst development following aphidicolin-induced replication stress

In the initial study, prolonged exposure (16 hours) to 1.4 µM aphidicolin resulted in cleavage arrest with no blastocyst formation in the uniform aphidicolin-treated group (0/30, 0%) compared to 29/30 (97%) blastocyst formation in the control group. This finding indicates a strong exposure-duration-dependent effect under the conditions tested. In the main trial, limited exposure (four hours) to 1.4 µM aphidicolin resulted in blastocyst formation rates of 26/30 (87%) in the control group, 22/30 (73%) in the pseudo-mosaic group, and 27/30 (90%) in the uniform aphidicolin-treated group. The OR for blastocyst formation was 2.36 (95% CI, 0.63-8.92) for control versus pseudo-mosaic embryos and 0.722 (95% CI, 0.147-3.55) for control versus uniform-treatment embryos. Blastocyst expansion and quality were also similar among the three cohorts (Figure 2).

**Figure 2:**
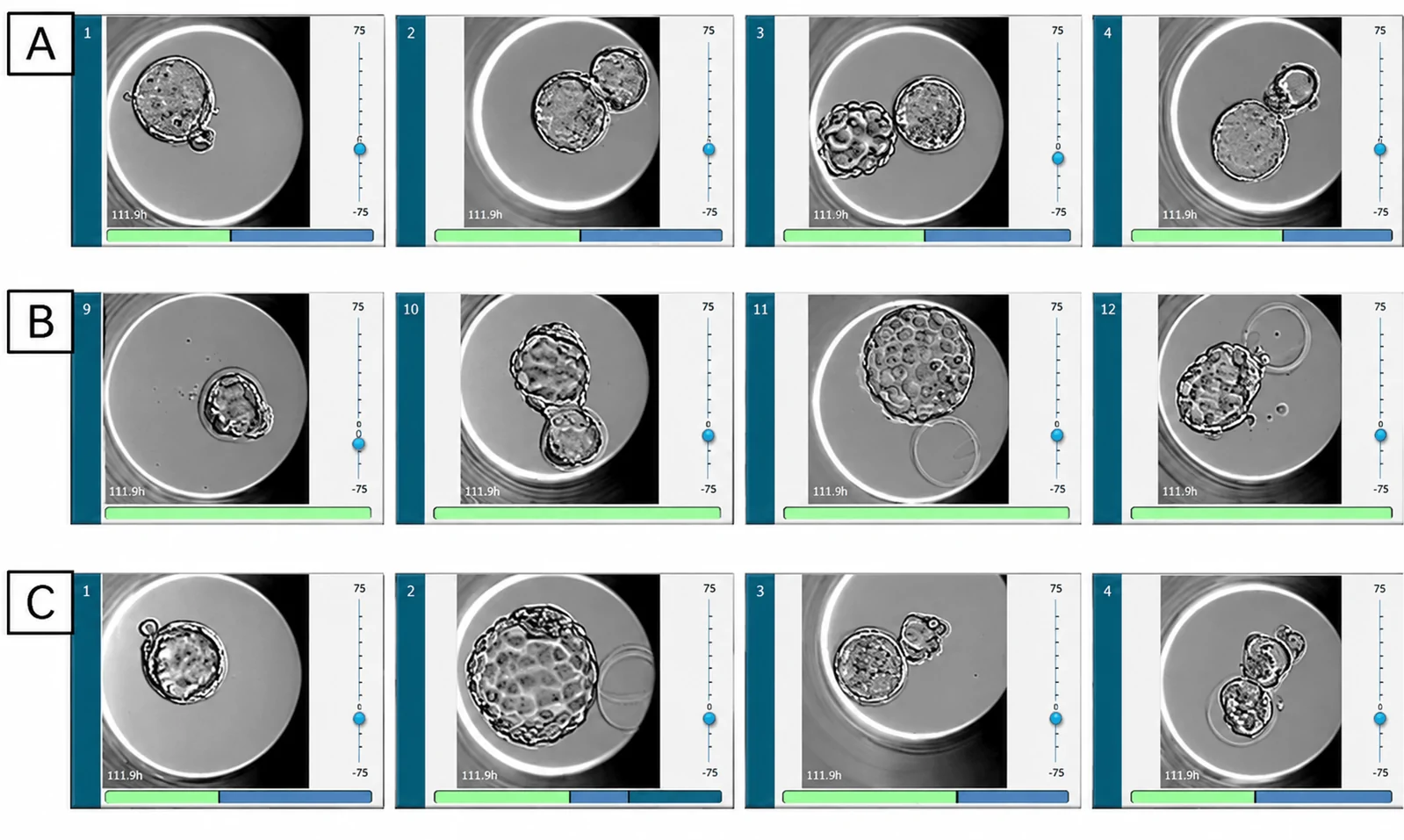
Time-lapse still images representative of blastocysts derived from thawed, cryopreserved, four-cell mouse embryos grown in vitro. (A) Untreated control group. (B) Pseudo-mosaic group demonstrating the incorporation of aphidicolin-treated cells. (C) Uniform aphidicolin-treated group. Image Credits: This figure was created with screenshots from the time-lapse incubator (Embryoscope+, Vitrolife Group, Gothenburg, Sweden).

Differential gene expression profiles differ between pseudo-mosaic and uniform-treated embryos

In the main study, differential expression analysis identified 791 differentially expressed genes (DEGs) in the control versus pseudo-mosaic comparison and 33 DEGs in the control versus uniform aphidicolin-treated comparison at an FDR-adjusted p-value ≤ 0.05. Of these, 349 and 14 genes, respectively, met the more stringent FDR-adjusted p-value threshold ≤ 0.01. Thus, a larger number of genes met the predefined criterion for differential expression in the control versus pseudo-mosaic comparison. However, because these DEG counts were derived from separate pairwise comparisons with the control group, the difference in the number of DEGs (791 versus 33) was not itself subjected to statistical testing and should be interpreted descriptively rather than as evidence of a significantly greater overall transcriptomic response. A direct DESeq2 comparison between the pseudo-mosaic and uniform aphidicolin-treated groups identified 45 DEGs at an FDR-adjusted p-value ≤ 0.05, of which 14 met the more stringent threshold of ≤0.01, demonstrating statistically supported gene-level differences between the two treatment groups. When the subset of 33 DEGs identified in the control versus uniform aphidicolin-treated comparison was examined across all three cohorts, approximately half were upregulated and half were downregulated in the uniform-treated group, whereas the pseudo-mosaic group exhibited a different pattern of expression (Figure 3A). Significant enrichment was observed in pathways related to RNA polymerase II cis-regulatory region sequence-specific DNA binding, transcription cis-regulatory region binding, and transcription regulator activity [28] (Figure 3B).

**Figure 3:**
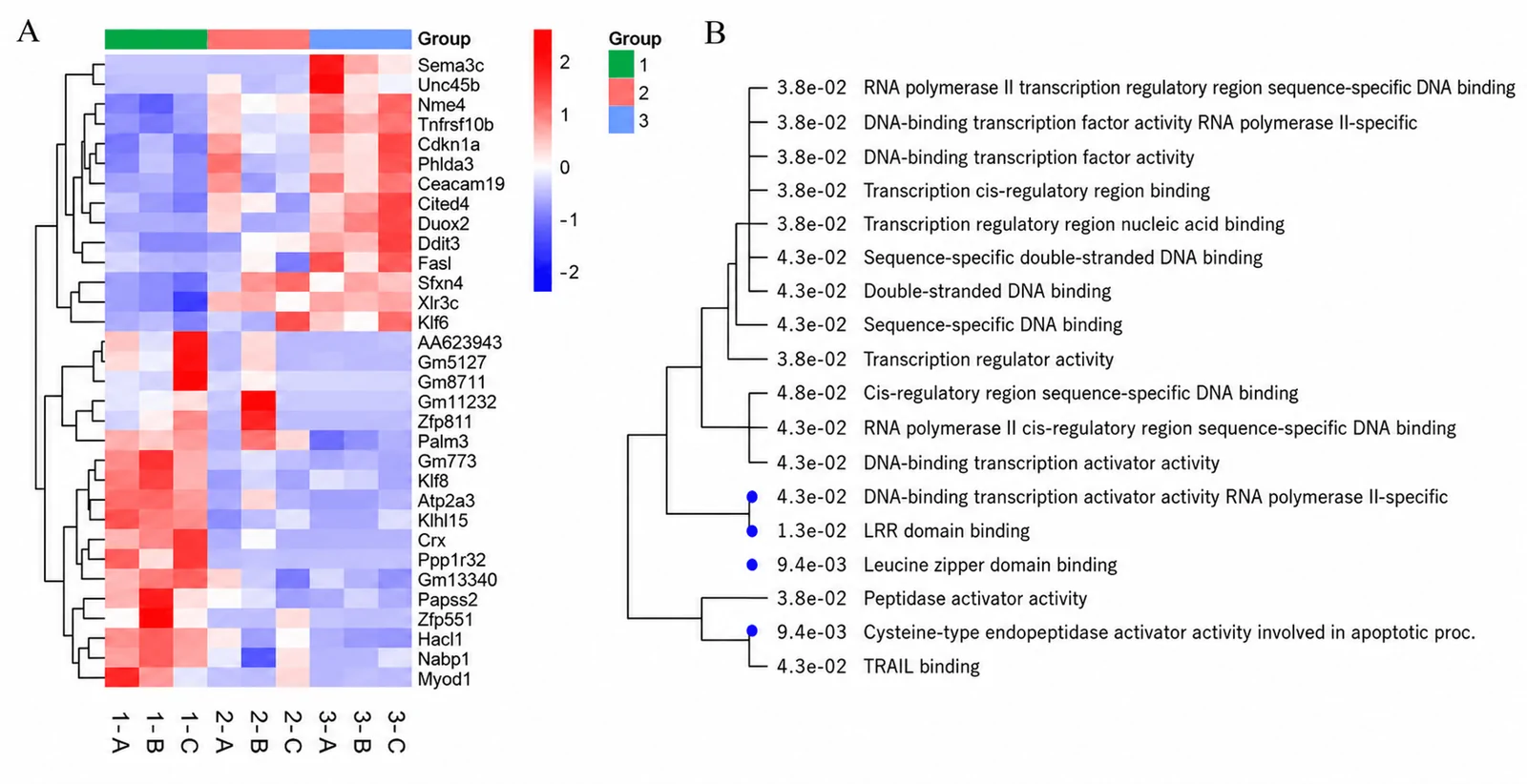
Differential gene expression in embryos treated with aphidicolin, an inhibitor of DNA polymerases. (A) Heatmap comparing DEGs across control (Group 1), pseudo-mosaic (Group 2), and uniform aphidicolin-treated (Group 3) cohorts. (B) Major molecular function pathways associated with represented DEGs.

Dichotomous gene pathway enrichment in two aphidicolin-treated groups

Pathway-enrichment analyses of the uniform aphidicolin-treated and pseudo-mosaic groups revealed differing patterns of enriched biological functions despite similar morphological development. In the uniform aphidicolin-treated group, DEGs were more commonly linked to transcriptional regulation, protease activation and apoptosis, and specific molecular interactions between DNA and proteins. These pathways included DNA-binding transcription activator activity and RNA polymerase II-specific DNA-binding transcription factor activity. Both upregulated and downregulated DEGs were identified in the uniform aphidicolin-treated group compared with the control group (Figure 4A). Upregulated genes shared themes related to cellular stress response, apoptosis, and gene regulation. Ddit3, a transcription factor associated with endoplasmic reticulum stress, was significantly upregulated in this model [29] (Figures 4B, 4C). Genes such as Myod1 and Crx, which are linked to differentiation and development, were downregulated in the uniform aphidicolin-treated group [28-30].

**Figure 4:**
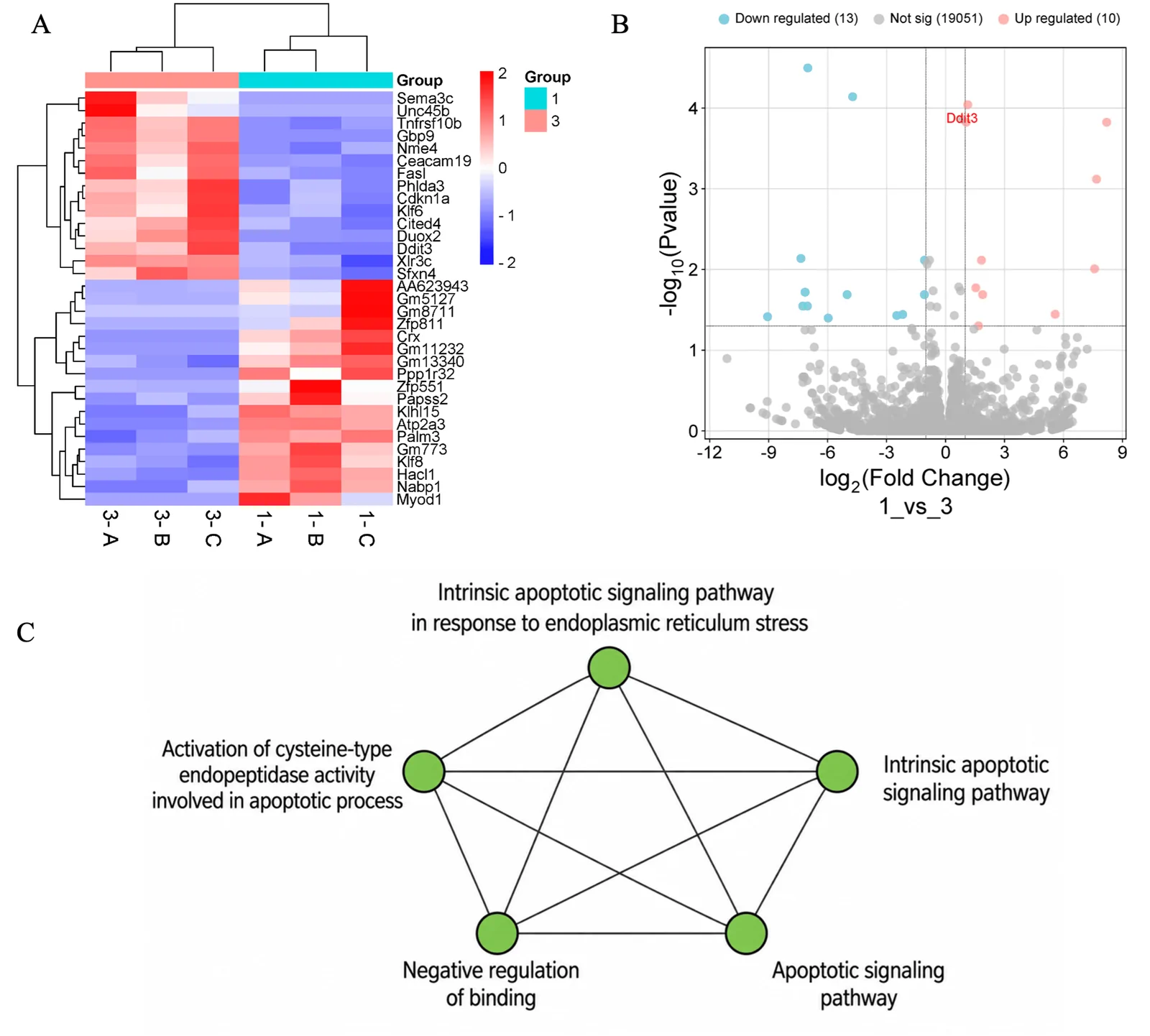
Differential gene expression and enriched pathways in uniform aphidicolin-treated embryos. (A) Heatmap comparing DEGs of uniform aphidicolin-treated embryos and the control group. (B) Volcano plot showing differential gene expression, including Ddit3. (C) Significantly enriched biological process pathways involving the 33 DEGs in the uniform aphidicolin-treated cohort.

The pseudo-mosaic group had numerous significantly enriched pathways, including pathways involving protein synthesis and degradation, molecular transport and energy production, and cellular stress responses (Figure 5). Collectively, these functions are consistent with changes in core cellular activities such as gene expression, protein homeostasis, energy metabolism, and cellular defense mechanisms [28-30]. These pathway-level differences may reflect compensatory proliferation, redundancy in critical cellular functions, and/or developmental plasticity; however, these interpretations remain hypotheses because genomic status and lineage fate were not directly measured in this study.

**Figure 5:**
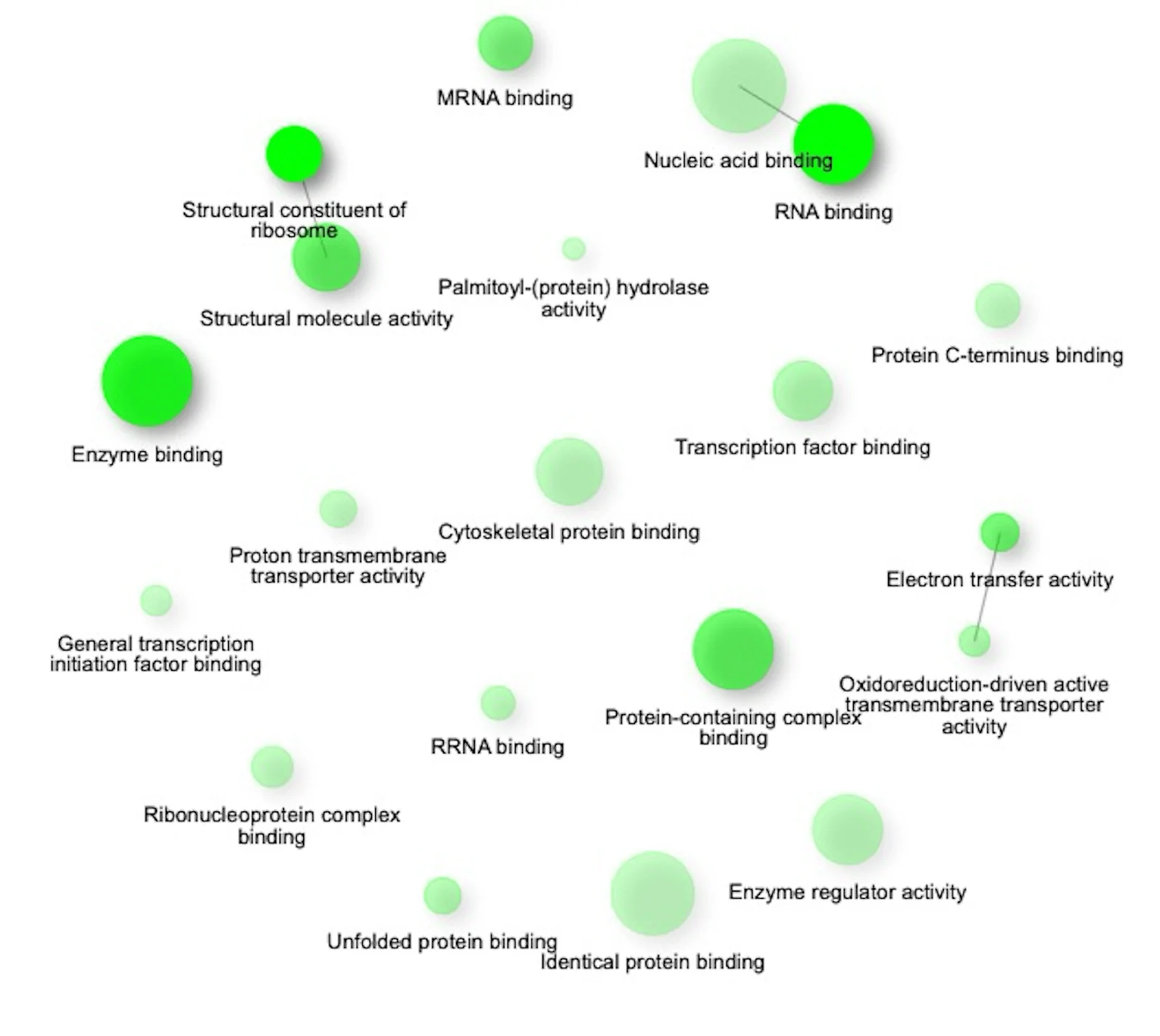
Enriched pathways in pseudo-mosaic embryos. Connected pathways share 20% or more genes. Darker circles represent more significantly enriched gene sets, while bigger circles represent larger gene sets. Image Credits: This figure was created using experimental data and running it through ShinyGO 0.80 (South Dakota State University).

Differential gene expression was observed in 791 genes in the pseudo-mosaic group, with approximately two-thirds upregulated (Figure 6A). The upregulated genes were mainly associated with energy metabolism, protein translation, protein folding, cell-cycle regulation, DNA repair, stress response, and apoptosis. In contrast, downregulated genes primarily contributed to gene regulation, cell signaling, cytoskeletal development, cellular structure, and development and differentiation. Nanog and Lin28a, which are important in stem-cell maintenance and differentiation, were downregulated in this group [30] (Figure 6B). Overall, these genes participate in pathways important for cell survival, growth, and specialization. The observed differential-expression patterns are consistent with altered cellular responses to replication stress in the pseudo-mosaic embryos; however, their functional significance remains uncertain, and evidence of adaptive or self-corrective mechanisms would require direct genomic and lineage-level validation.

**Figure 6:**
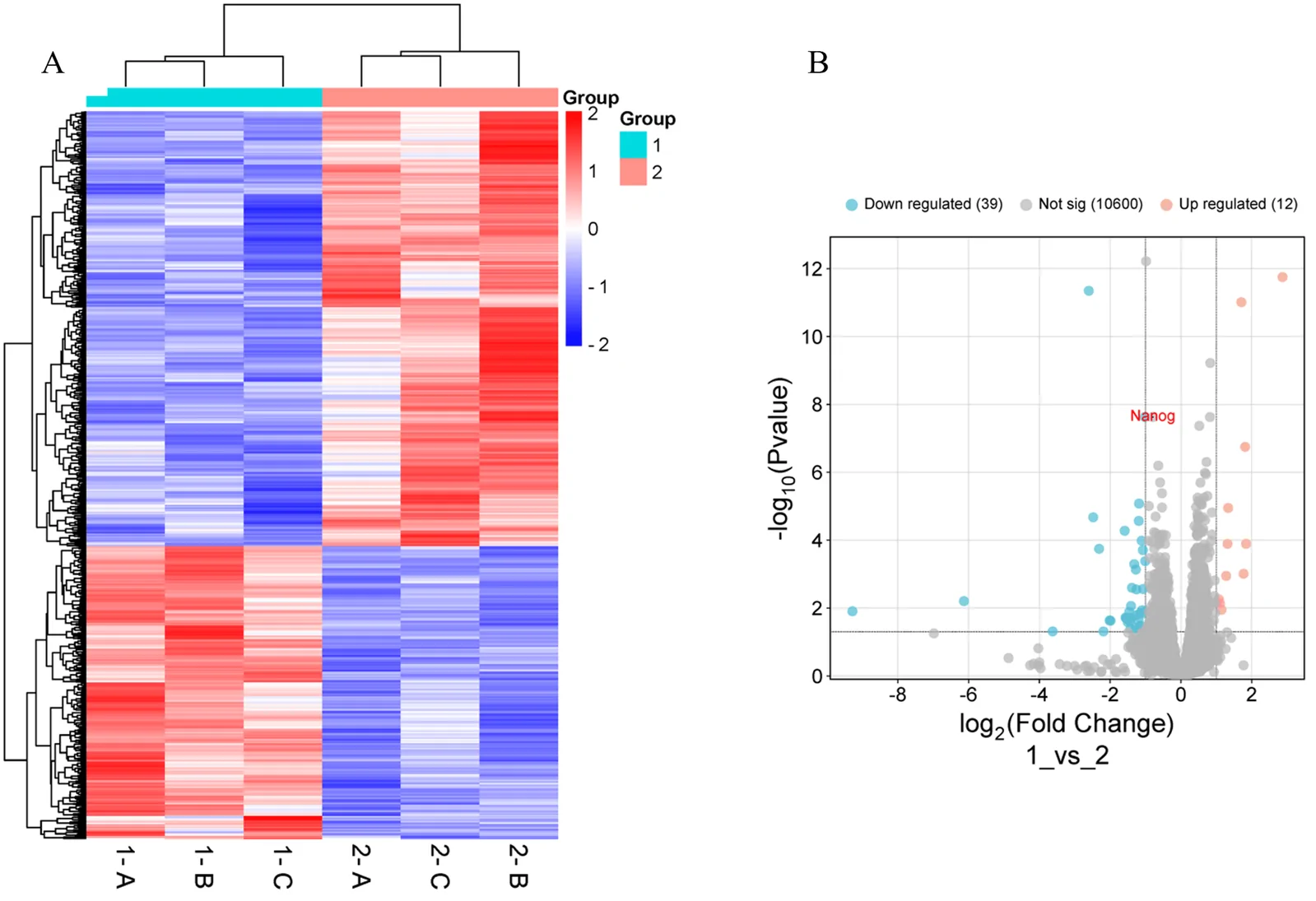
DEGs in pseudo-mosaic embryos (Group 2) compared with untreated control embryos (Group 1). (A) Heatmap showing approximately two-thirds of the DEGs are upregulated and one-third are downregulated in pseudo-mosaic embryos. (B) Nanog, downregulated in pseudo-mosaic embryos, is involved in development and differentiation.

## Discussion

This study compared differential gene expression in intact embryos exposed uniformly to aphidicolin with pseudo-mosaic embryos in which two of four blastomeres were removed, exposed to aphidicolin, and then reinjected into the corresponding embryo. All 30 treated pseudo-mosaic embryos demonstrated reincorporation of the manipulated blastomeres. However, because genomic testing and lineage tracing were not performed, reincorporation cannot be assumed to demonstrate persistence of a genetically abnormal lineage. The model should therefore be interpreted as a comparison between uniform and spatially restricted aphidicolin-induced replication stress rather than as direct confirmation of chromosomal mosaicism.

The developmental findings are consistent with previous evidence that early embryos can tolerate some forms of chromosomal or cellular stress without obligatory failure at the blastocyst stage. Mashiko et al. reported that chromosome-segregation errors during early cleavage in mouse embryos did not necessarily result in developmental failure after blastocyst formation [4]. Similarly, Bolton et al. demonstrated in a mouse model of chromosome mosaicism that aneuploid cells can undergo lineage-specific depletion while embryos retain developmental potential [9]. In the present study, four hours of aphidicolin exposure did not significantly reduce blastocyst formation in either treated cohort, despite clear transcriptomic differences. Together, these observations support the concept that morphological progression to the blastocyst stage does not necessarily indicate the absence of underlying cellular or molecular stress.

The contrast between the 16-hour preliminary exposure and the 4-hour main-study exposure also agrees with earlier work showing that the effects of cell-cycle-arrest agents on mouse embryos depend on both concentration and exposure duration. Samaké and Smith found that aphidicolin-mediated cleavage arrest became increasingly detrimental with prolonged exposure, with longer treatment intervals associated with embryo lethality and impaired development [23]. Our finding of 0/30 (0%) blastocyst formation following 16 hours of exposure, compared with preservation of blastocyst formation following 4 hours of exposure, is consistent with an exposure-duration-dependent toxicity threshold. Because the aphidicolin concentration was held constant between these experiments, the present data should not be interpreted as demonstrating a dose-response relationship.

The broader transcriptional response observed in the pseudo-mosaic group is also relevant to proposed mechanisms of embryonic adaptation. The 791 DEGs identified in pseudo-mosaic embryos, compared with 33 in uniform aphidicolin-treated embryos, suggest that cellular discordance may generate a more complex transcriptional environment than uniform replication stress. Enrichment of pathways related to metabolism, protein homeostasis, stress response, DNA repair, apoptosis, and cell-cycle regulation is compatible with a coordinated adaptive response. By contrast, the uniform-treated group showed a more restricted profile involving transcriptional regulation and stress-associated signaling, including upregulation of Ddit3. These findings do not establish a specific self-correction mechanism, but they provide candidate pathways for future mechanistic investigation.

Clinical and human-model studies provide additional context for these observations. In a prospective non-selection trial, Capalbo et al. reported equivalent live-birth and miscarriage rates among euploid, low-grade mosaic, and medium-grade mosaic blastocysts, with no detected mosaicism or uniparental disomy in the resulting pregnancies or newborns [25]. Yang et al., using human embryo and gastruloid models, demonstrated preferential depletion of aneuploid cells and showed that a relatively small euploid population could compensate for developmental deficits through mechanisms involving apoptosis [26]. Although the present mouse model differs substantially from naturally occurring human embryonic mosaicism, the distinct transcriptomic response observed after restricted blastomere exposure is directionally consistent with the broader concept that mixed cell populations may engage cell-survival, cell-selection, or compensatory programs that are not required when stress is distributed uniformly. These observations provide conceptual context rather than evidence of direct mechanistic equivalence, as naturally occurring human mosaicism, gastruloid models, and experimentally induced replication stress in mouse embryos differ substantially in developmental context, origin of cellular abnormalities, and experimental design.

The present results should therefore be viewed as hypothesis-generating rather than as evidence that aphidicolin-treated embryos undergo definitive chromosomal self-correction. Future studies combining single-embryo or single-cell RNA sequencing with direct genomic characterization, lineage tracing, and time-course sampling would help determine whether the pathways identified here correspond to selective elimination of damaged cells, compensatory proliferation of unaffected cells, altered lineage allocation, or other adaptive processes. Such approaches would also permit direct correlation between the magnitude and type of genomic abnormality and the transcriptional response.

An important limitation of this study is that aphidicolin-induced replication stress does not reproduce all mechanisms responsible for naturally occurring embryonic aneuploidy or mosaicism, which commonly result from chromosome-segregation errors rather than generalized pharmacologically induced replication stress. In addition, genomic testing was not performed to confirm the extent or chromosomal distribution of aphidicolin-induced abnormalities, and reincorporation of treated blastomeres does not establish that those cells subsequently generated a persistent abnormal lineage. Transcriptomic analysis was performed on pools of 10 embryos per biological replicate. Although pooling was necessary to obtain sufficient RNA for analysis, it may influence both the apparent magnitude and detection of differential gene expression by averaging transcriptional signals across embryos. This approach may obscure embryo-to-embryo heterogeneity, attenuate biologically meaningful responses present in only a subset of embryos, or allow strongly expressed signals from individual embryos to disproportionately influence the pooled expression profile. Accordingly, the present analysis cannot determine whether the observed expression differences were uniformly present across embryos within each group and does not permit direct correlation of individual-embryo genomic status, morphology, and transcriptomic response. The relatively small number of biological replicates further supports interpreting these findings as preliminary. Future studies incorporating single-embryo or single-cell RNA sequencing together with genomic characterization will be necessary to determine whether the pathway differences observed here represent reproducible mechanisms of embryonic adaptation or self-correction.

## Conclusions

This preliminary study demonstrates that transient aphidicolin-induced replication stress elicits distinct transcriptomic responses depending on whether the insult is present uniformly throughout the embryo or confined to a subset of blastomeres. Pseudo-mosaic embryos exhibited substantially broader differential gene expression and enrichment of pathways associated with cellular stress responses, DNA repair, metabolism, protein homeostasis, and apoptosis despite maintaining blastocyst development. These findings demonstrate that embryos containing discordant cell populations exhibit distinct transcriptional responses compared with embryos exposed to uniform cellular stress. Further genomic, lineage-tracing, and single-embryo studies are needed to determine whether these responses contribute to embryonic adaptation, self-correction, or developmental resilience.
